# Energy and speleogenesis: Key determinants of terrestrial species richness in caves

**DOI:** 10.1002/ece3.3558

**Published:** 2017-10-24

**Authors:** Alberto Jiménez‐Valverde, Alberto Sendra, Policarp Garay, Ana Sofia P. S. Reboleira

**Affiliations:** ^1^ Grupo de Investigación de Biología del Suelo y de los Ecosistemas Subterráneos Departamento de Ciencias de la Vida Facultad de Biología Ciencias Ambientales y Química Universidad de Alcalá Alcalá de Henares Madrid Spain; ^2^ Servei de Patrimoni Historic Ajuntament de València Spain; ^3^ Departament de Geologia Universitat de València Burjassot Spain; ^4^ Departamento de Biologia & CESAM Universidade de Aveiro Aveiro Portugal; ^5^ Natural History Museum of Denmark (Zoological Museum) University of Copenhagen København Ø Denmark

**Keywords:** biodiversity patterns, caves, energy, hypogean, hypogene karst, speleogenesis

## Abstract

The aim of this study was to unravel the relative role played by speleogenesis (i.e., the process in which a cave is formed), landscape‐scale variables, and geophysical factors in the determination of species richness in caves. Biological inventories from 21 caves located in the southeastern Iberian Peninsula along with partial least square (PLS) regression analysis were used to assess the relative importance of the different explanatory variables. The caves were grouped according to the similarity in their species composition; the effect that spatial distance could have on similarity was also studied using correlation between matrices. The energy and speleogenesis of caves accounted for 44.3% of the variation in species richness. The trophic level of each cave was the most significant factor in PLS regression analysis, and epigenic caves (i.e., those formed by the action of percolating water) had significantly more species than hypogenic ones (i.e., those formed by the action of upward flows in confined aquifers). Dissimilarity among the caves was very high (multiple‐site β_sim_ = 0.92). Two main groups of caves were revealed through the cluster analysis, one formed by the western caves and the other by the eastern ones. The significant—but low—correlation found between faunistic dissimilarity and geographical distance (*r *=* *.16) disappeared once the caves were split into the two groups. The extreme beta‐diversity suggests a very low connection among the caves and/or a very low dispersal capacity of the species. In the region under study, two main factors are intimately related to the richness of terrestrial subterranean species in caves: the amount of organic material (trophic level) and the formation process (genesis). This is the first time that the history of a cave genesis has been quantitatively considered to assess its importance in explaining richness patterns in comparison with other factors more widely recognized.

## 1 INTRODUCTION

The subterranean domain encompasses numerous habitats, which, despite being largely unexplored, are more dominant across the entire earth than surface habitats (Culver & Pipan, 2009). Caves—natural subterranean spaces in the underground that are accessible to humans—have traditionally been prioritized for biodiversity research as opposed to other subterranean habitats like, for instance, the mesovoid shallow substratum (Culver & Pipan, 2009; Jiménez‐Valverde et al., 2015); nevertheless, our knowledge of the biodiversity of the subterranean domain is markedly incomplete and has strong geographical biases (Culver & Holsinger, 1992; Gibert & Deharveng, 2002; Deharveng et al., 2009; Culver, Trontelj, Zagmajster, & Pipan, 2013). However, given the interest that subterranean biodiversity raises from evolutionary, ecological, biogeographical, and taxonomical standpoints, this diversity should be prioritized for conservation efforts (Romero, 2009; Culver & Pipan, 2009; White & Culver, 2012). Hence, understanding subterranean biodiversity patterns is crucial to establish measures that protect subterranean ecosystems from human‐induced global change (Christman & Zagmajster, 2012; Reboleira, Borges, Gonçalves, Serrano, & Oromí, 2011).

The diversity of terrestrial cave‐dwelling species tends to covary with the different geomorphological features found in each cave, as these attributes condition the abiotic environment (Culver & Pipan, 2010). A cave′s length has been repeatedly stressed as a key determinant of species richness, probably due to its direct influence on both the amount and diversity of microhabitats available (Culver, Christman, Šereg, Trontelj, & Sket, 2004; Kováč, Parimuchová, & Miklosová, 2016). Also, presumably, a cave′s length might be positively related to isolation, stability, and its conservation state. The altitude of the entrance also seems to be an important factor as it is directly related to temperature and productivity (Culver et al., 2004; Christman et al., 2016; Kováč et al., 2016), and species richness usually positively correlates with the amount of supplied energy (Hüppop, 2012). The size of the entrance might also correlate with the amount of organic material that goes inside the cave, as well as with the stability of the environment (Pellegrini, Aguiar, Sales, & Ferreira, 2016). Likewise, the availability of nutrients inside the cave is closely related to the presence of water, which is a factor that influences the physical and chemical features of the subterranean environment (Culver & Pipan, 2009). Temperature, productivity, stability, and the diversity of habitats have been widely recognized as important enhancers of species richness in surface terrestrial ecosystems (Whittaker, Willis, & Field, 2001), which might also be the case in the subterranean domain (Culver et al., 2006).

However, caves are not isolated entities. Caves are immersed in a karst system, and the length of its cavities may reflect the degree of development of that karst, which directly affects the volume of habitat that is available (Christman & Culver, 2001; Culver, Christman, Elliott, Hobbs, & Reddell, 2003; Culver et al., 2004, 2006; Niemiller & Zigler, 2013). On the other hand, caves (at least partially) could be considered ecotones that connect the surface and deep subterranean environments (Prous, Ferreira, & Martins, 2004; Moseley, 2009). Thus, surface variables such as temperature and precipitation have been identified as determinants of the presence of subterranean fauna probably due to their direct relationship with surface productivity (Christman et al., 2016). Landscape‐scale variables related to land use have also been pointed out as significant determinants in subterranean biodiversity patterns as they condition the influx of nutrients that seep into the underground (Pellegrini et al., 2016).

Another important factor that needs to be kept in mind in order to understand biodiversity patterns in caves is their speleogenesis history (Sendra et al., 2014). Contrary to the traditional epigenic karstification process, the formation of hypogenic caves occurs due to ascending corrosive fluxes under confined conditions (Klimchouk, 2007, 2009; Kimchouk, Ford, Palmer, & Dreybrodt, 2000; Klimchouk & Ford, 2009). The fact that hypogenesis occurs with poor or no connection to the surface due to confining layers of nonkarstificable rocks implies the late colonization of caves; this is only possible when the system connects to the surface or to other subterranean habitats as a result of the erosion of the confining layers (Sendra et al., 2014). Some decades ago, hypogenic karst was considered exceptionally rare, but now it is well known and is accepted as being present in many karst regions around the world (Klimchouk, 2007; Klimchouk & Ford, 2009; Kimchouk et al., 2000; Chavez & Reehling, 2016). It has been argued that the speleogenesis process may be the culprit in the scarcity of subterranean‐adapted fauna in certain environmentally suitable cave systems (Sendra et al., 2014).

The aim of this study was to unravel the role played by speleogenesis mode, landscape‐scale variables, and geophysical factors in the determination of species richness in caves. To achieve this, the biological inventories of 21 well‐studied caves in the southeastern Iberian Peninsula were compiled and partial least square (PLS) regression analysis was used to assess the relative importance of the different explanatory variables. The caves were grouped according to their species composition similarity, and the effect that spatial distance could have on similarity was also studied using correlation between matrices.

## 2 MATERIAL AND METHODS

### 2.1 Study area

**Figure 1:**
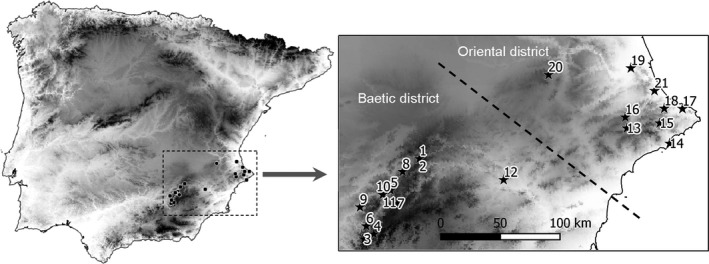
Region and caves considered in this study. The background represents elevation; the darker the color, the higher the altitude. Numbers correspond with caves as shown in Table 1 and Figure 3. Note that caves #10 and #11, as well as #1 and #2, are very close to one another, and they appear as just two points instead of four in the figure. A dashed line in the right panel separates the Baetic and Oriental biogeographical regions (Bellés, 1987)

This study was carried out in the northeast area of the Baetic Mountain Range (the Prebaetic System), which is located in the south of the Iberian Peninsula (Figure 1). The Iberian Prebaetic System extends over approximately 55,000 km^2^ throughout several mountain ranges, from the Guadalquivir depression to the Mediterranean shore (Ayala et al., 1986). The western limit of the Prebaetic System is outlined by a geological fault, and then, the domain prolongs northeast until the Valldigna valley, in the frontier with the Iberian Mountain Range. The southern limit of the study area extends across the so‐called Sub‐Baetic zone, where marine Mesozoic materials are folded in thrust nappes and sprinkled with volcanic materials (Fig. S1 in Appendix S1). Overall, the Prebaetic System is characterized by its alternation of carbonate rock massifs from the Jurassic and Cretaceous periods with marlstone depressions (Durán, López, & Vallejo, 1998), sprinkled with hundreds of epigenic caves and a few hypogenic ones. Twenty‐one caves were selected for this study (Table 1), of which three have an hypogenic speleogenesis: the Autopista and Far caves (Sendra et al., 2014) and the Puerto cave (Ros & Llamusí, 1989).

**Table 1:** Caves considered in this study. Numbers between brackets correspond with the caves shown in Figure 1 and Figure 3. Water presence has four ordered levels (1, occasional presence of areas of water or humid spots; 2, presence of scattered areas of water in the form of more or less continuous drops; 3, presence of permanent pools throughout the cave; 4, presence of rivers or streams), the tropic level has three ordered levels (1, oligotrophic, not having organic material; 2, mesotrophic, scattered organic material present along the cave; 3, meso‐eutrophic, with accumulations of organic material present along the cave), and genesis has two categories (0, epigenic; 1, hypogenic)

Cave name	Altitude (m.s.n.m.)	Size of entrance (m^2^)	Linear extension (m)	Water	Trophic level	Genesis
Cueva de los Chorros (1)	1,122	300	30,000	4	1	0
Cueva del Farallón (2)	1,250	1.2	600	3	2	0
Cueva‐Sima de los Ladrones (3)	1,570	1	315	2	2	0
Cueva Secreta del Poyo Manquillo (4)	1,500	3	296	2	2	0
Sistema de la Murcielaguina (5)	1,085	10	4,500	2	3	0
Cueva Secreta del Sagreo (6)	1,000	1	236	2	3	0
Cueva del Javalí (7)	1,520	1.5	190	2	3	0
Sima de los 30 Años (8)	1,383	4	340	2	2	0
Cueva de la Morciguilla (9)	700	0.5	480	2	2	0
Sima del Campamento (10)	887	15	538	2	2	0
Sima de la Tubería (11)	930	0.5	65	2	2	0
Cueva del Puerto (12)	495	0.5	5,000	2	1	1
Cova Joliana (13)	653	1	1,100	3	2	0
Cova del Far (14)	120	0.8	1,100	2	2	1
Cova del Somo (15)	860	4.5	1,318	2	2	0
Cova de les Meravelles (Cocentaina) (16)	1,070	0.5	157	2	2	0
Cova de la Punta de Benimaquía (17)	60	12	208	2	3	0
Cova Sant Joan (18)	250	0.8	15	1	2	0
Cova de les Meravelles (Alzira) (19)	60	8	60	2	3	0
Cueva Negra (20)	1,180	72	380	1	1	0
Cueva de la Autopista (21)	90	8	8,000	2	1	1

### 2.2 Biological data

Twenty‐one caves, which have been historically surveyed intensively using direct observation and pitfall traps, were chosen (see Table S1 in Appendix S1). Most of the faunistic data have already been published in the biospeleological literature (see References in Appendix S1), with the exception of a few unpublished cases. Due to the heterogeneity of the surveys and to the lack of information needed to evaluate the completeness of the inventories, it is impossible to statistically judge their reliability or to standardize them to make them comparable. However, these 21 caves have been sampled more intensively in the region, all with pitfall trapping, and thus, to the best of our knowledge, they represent the best set of available inventories to work with. The database only includes terrestrial subterranean fauna from each of the cave′s deepest zone, that is, troglophile and troglobiont species (93 and 33 species, respectively) sensu Schiner (1854) and Racovitza (1907). The 126 subterranean species and two subspecies in this inventory belong to invertebrates, mostly arthropods, plus five species of molluscs (see Table S1 in Appendix S1). Among arthropods, there are as follows: 29 species of Arachnida, 13 of Myriapoda, 13 Crustacea Oniscidea, 21 Collembola, 4 Diplura, and 41 Insecta, mostly Coleoptera (31 species).

### 2.3 Explanatory variables

For each cave, six local biogeophysical variables were obtained from the literature or from personal observations of the authors (Table 1): (1) linear extension, (2) altitude above sea level, (3) size of the entrance, (4) water availability (presence of rivers or streams; presence of permanent pools; presence of scattered areas with continuous water drops; occasional presence of areas with water or humid spots), (5) trophic level (oligotrophic, not having organic material; mesotrophic, scattered organic material present along the cave; meso‐eutrophic, with accumulations of organic material present along the cave), and (6) the (speleo)genesis of the cave (epigenic *versus* hypogenic). Additionally, at the landscape level, eight variables (two related to land use, one related to geology, and five related to climate) were considered. The percentages of natural vegetation (forest, shrub, and herbaceous associations) and agricultural land within a 500‐m buffer around each cave entrance were extracted from the European Corine Land Cover 2006 raster GIS layer at 100‐m resolution (European Environment Agency, http://eea.europa.eu). In the case of natural vegetation, Corine categories 3.1.1–3.2.4 were considered and grouped together; in the case of agriculture, categories 2.1.1–2.4.4 were considered and merged. The percentage of karst area (limestone, gypsum, and clastic formations) in a 15‐km buffer around each cave entrance was calculated using the digital version of the karst map of Spain (IGME, 1986) provided in vector format by the Spanish Geological and Mining Institute (Instituto Geológico y Minero de España, http://info.igme.es/cartografia). For each cave entrance, five climatic variables—mean temperature, mean minimum temperature, mean maximum temperature, mean number of days with temperatures ≤0°C, mean precipitation—were extracted from raster climatic models at 1‐km resolution provided by the Spanish Meteorological Agency (Agencia Estatal de Metoerología, http://www.aemet.es/es/serviciosclimaticos/datosclimatologicos/atlas_climatico).

GIS analyses were conducted in QGIS Wien Desktop version 2.8.1 (http://www.qgis.org).

### 2.4 Data analysis

To study the relationship between species richness (*S*) and the potential explanatory variables, a partial least square (PLS) regression analysis was applied, given the low sample size (*n *=* *21 caves) and the relatively large number of intercorrelated predictors (Carrascal, Galván, & Gordo, 2009). PLS regression is a multivariate technique that finds latent orthogonal components as linear combinations of predictors and maximizes the explained variance in the dependent variable (see Carrascal et al., 2009 and references therein). The linear extension and size of the entrance were log‐transformed prior to the analyses. After the number of significant latent components was found via cross‐validation (Geladi & Kowalski, 1986), the presence of atypical observations was inspected using Hotelling′s *T* ^2^ values (Jackson, 1991). A jackknife procedure (21 PLS regression models were fitted, using 20 observations on each occasion after excluding one cave in each turn) was applied to assess the robustness of the weights of the variables in the retained latent components. A simple linear regression (LR) model of *S* as a function of the retained PLS regression components was fitted, and longitude was added to test whether a geographical W‐E gradient could explain the extra amount of variance. The presence of outliers, heteroscedasticity, and a lack of normality in the residuals of the LR model was inspected, and the percentile bootstrap method proposed by Wilcox (1996) was used to compute confidence intervals for the LR parameters in case of a violation of homoscedasticity. Analyses were conducted in R version 3.1.1 (R Development Core Team, 2014) using the “plsdepot” version 0.1.17 package (Sánchez, 2016) and the “lsfitci” function (Wilcox, 2012).

Using the presence/absence community matrix, and to get an overall value of fauna dissimilarity among the 21 caves, the multiple‐site distances based on the Simpson index (β_sim_, which measures dissimilarity due to turnover—that is, it is independent of species richness differences) was computed (Baselga, Jiménez‐Valverde, & Niccolini, 2007). A faunistic distance matrix based on β_sim_ was also computed, and a Mantel test based on Pearson's product‐moment correlation was used to test for correlation between this matrix and a geographical distance matrix. The β_sim_ ‐based faunistic distance matrix, together with Ward's method as linkage rule (which minimizes the difference between the sum of the squared distances of cases and the mean values of the clusters to which they are assigned (Legendre & Legendre, 1998)) was used to group caves according to their taxonomic resemblance. Analyses were conducted using the “vegan” version 2.3.5 (Oksanen et al., 2016), “ecodist” version 1.2.9 (Goslee & Urban, 2015), and “betapart” version 1.3 (Baselga, Orme, Villeger, De Bortoli, & Leprieur, 2015) packages for R.

## 3 RESULTS

One significant PLS latent component was obtained accounting for 44.3% of the variation in species richness. This component was positively related to the trophic level, the most important variable accounting for 44.7% of its informative content. The latent component was negatively related to the genesis mode, which accounted for 24.0% of the informative content—that is, epigenic caves had significantly more species than hypogenic ones (Table 2). The latent component was also positively related to the amount of agricultural land, although the relevance of this variable was low in comparison with previous ones (accounting for only 7.7% of the informative content of the component, Table 2). Hotelling′s *T* ^2^ values identified one possible influential observation (see Fig. S2 in Appendix S1), and the jackknife procedure showed stability in the weights for trophic level and genesis mode but not for the amount of agriculture land (Table 2 and Fig. S3 in Appendix S1).

**Table 2:** Results of the partial least square (PLS) regression analysis. The variables whose effect is larger than expected by chance, that is, those whose square weights are larger than 1/(number of explanatory variables) are indicated in bold‐type font

Explanatory variables	Correlation with component 1	Square weight in component 1	Range of jackknifed square weights
Karst area	0.323	0.046	0.015–0.088
Agriculture land	0.238	**0.077**	0.020–0.131
Natural vegetation land	−0.135	0.054	0.007–0.093
Mean maximum temperature	−0.392	<0.001	<0.001–0.014
Mean precipitation	0.596	0.041	0.008–0.091
Days with temperatures ≤ 0ºC	0.527	0.018	0.001–0.043
Mean minimum temperature	−0.494	0.026	0.002–0.065
Mean temperature	−0.466	0.010	<0.001–0.033
Altitude	0.442	0.006	<0.001–0.027
Size of entrance (log‐transformed)	−0.120	<0.001	<0.001–0.008
Linear extension (log‐transformed)	−0.502	0.030	0.002–0.093
Water	0.043	0.006	<0.001–0.056
Trophic level	0.786	**0.447**	0.382–0.501
Speleogenesis	−0.843	**0.240**	0.165–0.280

**Figure 2:**
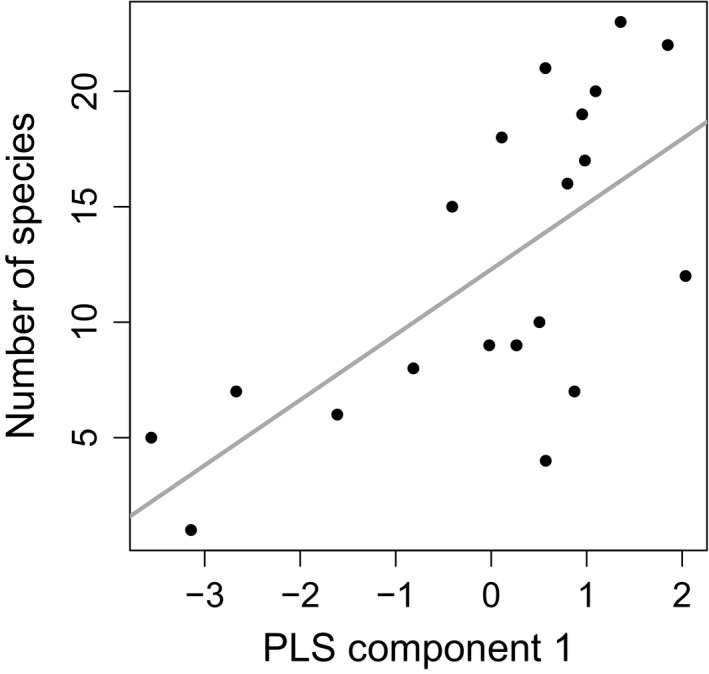
The relationship between the number of species and the position of each cave in component 1 of the partial least square (PLS) regression analysis

The LR model of *S* as a function of the latent component corroborated its statistical significance (β_1_ = 2.827, *SE* = 0.727, 95% CI = 1.305–4.349, *t *=* *0.388, *p* < .001; Figure 2). Residuals were normally distributed according to the Shapiro–Wilk test (W = 0.95, *p* = .336) (see also in Fig. S4 in Appendix S1), and there were no influential points (see Fig. S3 in Appendix S1). A problem of heteroscedasticity was apparent (see Fig. S4 in Appendix S1), but the 95% CI for the slope estimated using the Wilcox percentile bootstrap method was even narrower (1.529–4.192). Longitude did not explain any extra amount of variation in species richness, so there was no western–eastern pattern in the residuals that remained to be explained.

**Figure 3:**
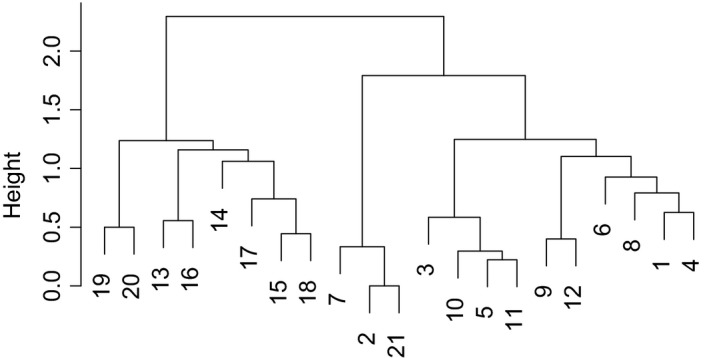
Dendrogram showing the faunistic similarity of caves using the Simpson index (β_sim_) as a distance measure and Ward′s method as a linkage rule. Numbers correspond with the caves shown in Table 1 and Figure 1

Dissimilarity among caves was very high, with a multiple‐site β_sim_ value of 0.92. The dissimilarity matrix based on β_sim_ showed a positive and significant correlation with the spatial distance matrix, although the strength of the relationship was low (*r* = .16, *p* = .016). The cluster analysis (Figure 3) showed two main groups of caves, one formed by the western caves and the other by the eastern ones (the Autopista cave—cave #21—appears in the western group, but this cave only has one species; see Figure 1). The effect of spatial distance on dissimilarity disappeared when two separate Mantel tests were run, one for each cluster of caves (excluding cave #21, *r* = −.08, *p* = .711 for the western group, and *r* = .21, *p* = .153 for the eastern group).

## 4 DISCUSSION

The Prebaetic System has experienced a continuous karstification process (i.e., limestone dissolution) since the end of the Miocene (Durán et al., 1998), currently resulting in a well‐developed karst system with subterranean spaces that have remained accessible to fauna colonization for an extended period of time. Furthermore, the whole Prebaetic System has kept a relatively stable and mild climate and has experienced a common paleogeographic history during this long period of time (López, 1989). Nowadays, the subterranean fauna of this region is composed by a flourishing variety of troglophile and troglobiont species: of the 128 taxa (found in the 21 caves), 33 are troglobionts, which clearly illustrates the relevance of this geographical area in the Iberian context for its subterranean biodiversity (see Sendra et al., 2011). The Prebaetic System is actually part of two different biogeographical regions, the Baetic and the Oriental (or Levant) districts (sensu Español in Bellés, 1987; Sendra et al., 2011). These bioregions were apparent in the cluster analysis (Figure 3), which clearly separated the eastern (Oriental) from the western (Baetic) caves (Figure 1). The relatively low alpha diversity (from 1 to 23 species, median = 10) contrasts with the high beta‐diversity found, corroborating a usual pattern in the subterranean domain probably caused by low connectivity among caves (i.e., high habitat patchiness) and/or a very low dispersal capacity of the species (Nekola & White, 1999; Culver & Sket, 2000; Malard et al., 2009; Niemiller & Zigler, 2013; Sánchez‐Fernández et al., 2016). It is remarkable that 53% of the species were found in just one cave (see Table S1 in Appendix S1); hence, the high species turnover found. In fact, it is not unexpected that the low importance that geographical distance had to account for dissimilarity in species composition (see also Zagmajster et al., 2014 for a case with groundwater crustaceans) ended up disappearing when the caves were split into the two bioregions. Pellegrini et al. (2016), working at a much lower spatial extent, also did not find any effect of geographical distance on species dissimilarity.

The results of this study show that, in the Prebaetic System, two main factors seem to be intimately related to terrestrial subterranean species richness within caves: the amount of organic material (trophic level) and the process of formation (genesis). Both variables are interrelated, and thus, it is not possible to distinguish their independent roles as, in this study, there are no hypogenic and nutrient‐rich caves; however, the trophic level arises as the most important determinant, with the highest weight in the PLS latent component. It is known that the amount of organic material acts as a limiting factor for the colonization of a subterranean environment (Pipan & Culver, 2013). Thus, caves in very cold or desert regions have a markedly depauperated or even inexistent fauna, and besides direct bioclimatic reasons (Howarth, 1980; Culver et al., 2006), some authors have argued that this may be caused by the fact that organic matter hardly ever reaches the deep layers in these environments (Sendra & Reboleira, 2014). As in caves nutrient input is allochthonous (Culver, 1982; Howarth, 1983; but also see, for instance, Sarbu, 2000 or Hutchins, Engel, Nowlin, & Schwartz, 2016), subterranean species richness variation is related to primary productivity at the surface, as suggested by the high biodiversity spots located in highly productive latitudinal bands (Culver et al., 2006; Gibert & Deharveng, 2002). Although other geophysical and landscape‐scale variables might be related to the amount of energy supply and, consequently, to biodiversity patterns (e.g., Christman et al., 2016), once this factor is directly taken into account, no other variables at the spatial scale of this study showed a significant effect on species richness. The only exception was the amount of agricultural land, which had a positive effect on species richness probably due to the percolation of nutrients. However, the instability of the weight of this factor in the jackknife procedure suggests caution concerning the positive effect of agriculture, especially when negative effects on the health of subterranean ecosystems have been observed (Di Lorenzo et al., 2014; Reboleira, Abrantes, Oromí, & Gonçalves, 2013).

**Figure 4:**
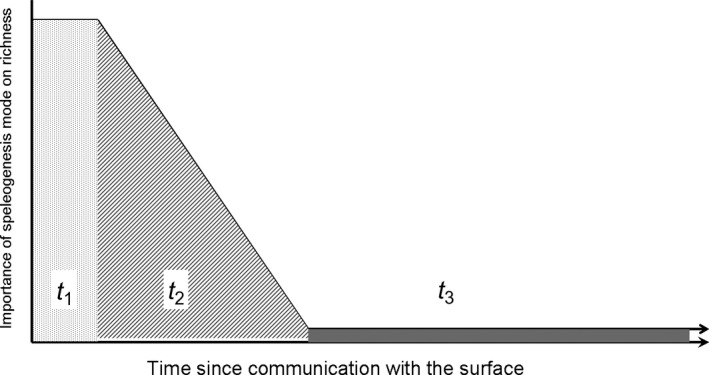
Conceptual model of the importance of speleogenesis mode evolution on the determination of species richness over time. Origin: a connection is established between the hypogenic system and the surface. In the first phase (*t*_1_), there has not been enough time for organic matter and species entrance into the cave. In the second phase (*t*_2_), nutrients begin to accumulate and species start to appear inside the cave; the relevance of the origin of the cave as a factor that controls species richness dilutes over time. Finally, in the last phase (*t*_3_), hypogenesis is no longer an important factor in explaining richness patterns. Other factors, such as the degree of connection with the surface, may interact with time and change the relative duration of the different phases

Although it has already been pointed out that the age of caves can explain subterranean richness figures (e.g., Poulson & Culver, 1969), to the best of our knowledge this is the first time that the history of the caves has, in some way, explicitly and quantitatively been considered to assess its relative importance in comparison with other more widely recognized factors. The ecological consequence for the existence of confining layers during the development of hypogenic systems is the isolation from the surface. This isolation stabilizes the climate, usually causes higher mean temperatures than on the surface due to geothermal anomalies, and prevents nutrients and species from entering hypogenic subterranean spaces (Sendra et al., 2014). In this way, faunal colonization can only occur when the confining barrier is disrupted and the hypogenic subterranean systems are able to connect with either the surface and/or with other epigenic subterranean systems (Sendra et al., 2014). The vast majority of accessible hypogenic caves are relicts, and most of them are already fossilized (Klimchouk, 2007), which means that they have been opened to the surface for a long time. Obviously, in these caves, hypogenic speleogenesis is no longer a relevant factor, as time has diluted its importance by allowing organic materials to enter and species to colonize the cave (Figure 4). For instance, this is the case in large hypogenetic caves in Brazil, which have been opened to the surface for millions of years (Auler, 2009), leading some authors to overly simplify and underestimate the role of speleogenesis (e.g., Trajano, Gallão, & Bichuette, 2016). On the contrary, speleogenesis is important in the cases of caves with recent openings to the surface (Figure 4). The three hypogenic caves of this study—Autopista, Far, and Puerto—have experienced different erosion histories: The Autopista cave was exposed thirty years ago as the result of the construction of a highway that cut through the confining layer (Sendra et al., 2014); natural erosion exposed the Far cave around a thousand years ago (Sendra et al., 2014); and finally, the Puerto cave, the only one harboring troglobiont species, has probably been open for much longer, as suggested by the almost complete disappearance of its confining layer (Fig. S5 in Appendix S1). But time is not the only important factor. The way the exposure happens is determinant; if the destruction of the confining layers occurs only in a few spots, then these caves will be poorly colonized by fauna due to poor communication with the surface, which is the case of some of the largest hypogenic caves in the world (Sendra et al., 2014).

There are two main limitations in subterranean‐biology research. One is the lack of accessibility to the subterranean environment, which means the whole micro‐cavern network is ignored and limits the studies to macro‐caverns (i.e., caves), and consequently makes extrapolation the only way to make inferences about the whole subterranean ecosystem (Culver et al., 2003, 2004; Graening, Slay, & Bitting, 2006; Kováč et al., 2016; Silva, Martins, & Ferreira, 2011; Sendra & Reboleira, 2012). The second limitation is the inability to obtain complete and reliable fauna inventories for single caves, single subterranean locations, or even entire regions (Schneider & Culver, 2004; Jiménez‐Valverde et al., 2015). In fact, it is possible that the inventories considered for this study were not complete or did not show a comparable degree of completeness, and this may be the reason why only less than a half (44.3%) of the amount of variation in species richness could be explained. One key issue that future faunistic studies of the subterranean domain should seriously consider is to report detailed data necessary for the evaluation of the inventories, such as the abundance of the species (not just presence/absence), number of traps, time spent on the survey, space sampled, or any other measure that could be useful in assessing sampling efforts. Yet, taking these limitations into account, this study has endeavored to discover the key determinants for the number of terrestrial subterranean species that are present in (i.e., have colonized) caves as an approach to better understanding the processes that operate between surface and phreatic levels, that is, in the whole vadose zone. Clearly, the amount of energy available in the system and the genesis mode of those systems are the two main factors conditioning species richness.

In future studies, in order to be able to quantify more precisely the weight of each factor, it is crucial to increase the number of caves. Twenty‐one was the maximum number of caves that were considered as having had a history of exploration sufficiently intensive so that their inventories could be considered, to some extent, reliable. The emergence in the cluster analysis of the two bioregions recognized in the literature suggests that the inventories are, at least, robust enough to obtain meaningful results. The other two issues that should be considered in future studies are as follows: (1) a way to better quantify the amount of energy that goes into the cave, and (2) explicitly including the amount of time that hypogenic systems have been in contact with the surface. These considerations would help improve the necessarily simplistic model depicted in Figure 4, which nevertheless, we hope will serve as a zero‐order approximation for understanding the role of history in the determination of subterranean biodiversity patterns.

## CONFLICT OF INTEREST

None declared.

## AUTHOR CONTRIBUTIONS

The four authors conceived the ideas; A.S. collected the biological data; A.S., P.G., and A.J. collected the descriptive variables; A.J. analyzed the data; A.J. and A.S. lead the writing; all authors contributed to the critical discussion and writing.
