# Supplementary material for: Energy and speleogenesis: Key determinants of terrestrial species richness in caves
Source: Ecol Evol. 2017 Oct 24;7(23):10207–15. doi: 10.1002/ece3.3558 (PMC5723612; doi:10.1002/ece3.3558)
Supplement: Supplementary file 1 [file ECE3-7-10207-s001.doc]

*Ecology and Evolution*

**Appendix S1**

**ENERGY AND SPELEOGENESIS: KEY DETERMINANTS OF TERRESTRIAL SPECIES RICHNESS IN CAVES**

Alberto Jiménez-Valverde, Alberto Sendra, Policarp Garay and Ana Sofia P. S. Reboleira

**Table S1** Species distribution in the studied caves. Number between brackets refers to the bibliographic reference of References in this Appendix. # unpublished data. * troglobiont species. ** troglophile species.

| TAXA | Cueva de los Chorros, Riópar | Cueva del Farallón, Riópar | Cueva-Sima de los Ladrones, Peal del Becerro | Cueva Secreta del Poyo Maquillo, Cazorla | Sistema de la Murcielaguina, Hornos | Cueva Secreta del Sagreo, La Iruela | Cueva del Javalí, Santiago de la Espada-Pontones | Sima de los 30 Años, Siles | Cueva de la Morciguilla, Villacarrillo | Sima del Campamento, Hornos | Sima de la Tubería, Hornos | Cueva del Puerto, Calasparra | Cova Joliana, Alcoi | Cova del Far, Alfàs del Pi | Cova del Somo, Castell de Castells | Cova de les Meravelles, Cocentaina | Cova Punta de Benimaquía, Dènia | Cova Sant Joan, Pego | Cova de les Meravelles, Alzira | Cueva Negra, Ayora | Cueva de la Autopista, Gandía |
| --- | --- | --- | --- | --- | --- | --- | --- | --- | --- | --- | --- | --- | --- | --- | --- | --- | --- | --- | --- | --- | --- |
| GASTROPODA: PULMONATA | | | | | | | | | | | | | | | | | | | | | |
| *Oxychilus (Ortizius) courquini* (Bourguignat, 1870) ** (14) |  |  |  |  |  |  |  |  |  |  |  |  | + |  |  |  |  |  |  |  |  |
| *Oxychilus (Ortizius) mercadali* Gasull, 1970** (#, 39) |  |  |  |  |  |  |  |  |  |  |  |  | + |  |  |  | + |  |  |  |  |
| *Oxychilus (Ortizius) rateranus* (Servain, 1880)** (24) |  |  |  | + |  |  |  |  |  |  |  |  |  |  |  |  |  |  |  |  |  |
| *Platyla polita* (Hartmann, 1840)** (#) |  |  |  |  |  |  |  |  |  |  |  |  |  |  |  |  | + | + |  |  |  |
| Suboestophora boscae (Hidalgo, 1869)** (#) |  |  |  |  |  |  |  |  |  |  |  |  |  |  | + | + |  | + |  |  |  |
| ARACHNIDA: ACARINA: ORIBATIDA | | | | | | | | | | | | | | | | | | | | | |
| *Damaeus gevi* Subias, 2012* (6, 25) |  | + |  |  | + |  |  |  |  | + | + |  |  |  |  |  |  |  |  |  |  |
| ARACHNIDA: OPILIONES | | | | | | | | | | | | | | | | | | | | | |
| *Dicranolasma soerensenii* Thorell, 1876** (39) |  |  |  |  |  |  |  |  |  |  |  |  |  |  |  |  | + |  |  |  |  |
| *Scotolemon* sp.** (16) |  |  |  |  |  |  |  |  |  |  |  |  |  | + |  |  |  |  |  |  |  |
| ARACHNIDA: PSEUDOESCORPIONES | | | | | | | | | | | | | | | | | | | | | |
| *Allochernes masi* (Navás, 1923)** (23) |  |  |  |  |  |  |  |  | + |  |  |  |  |  |  |  |  |  |  |  |  |
| *Chthonius (Ephippiochthonius)* *cazorlensis* Carabajal Márquez, García Carrillo & Rodríguez Fernández, 2001** (23) |  |  |  |  |  | + |  |  |  |  |  |  |  |  |  |  |  |  |  |  |  |
| *Chthonius (Ephippiochthonius) espanyoli* Zaragoza & Pérez, 2013* (23) |  |  |  | + |  |  |  |  |  |  |  |  |  |  |  |  |  |  |  |  |  |
| *Chthonius (Ephippiochthonius) giennensis* Zaragoza & Pérez, 2013* (23) |  |  | + |  |  |  |  |  |  |  |  |  |  |  |  |  |  |  |  |  |  |
| *Chthonius (Ephippiochthonius) perezi* Carabajal Márquez, García Carrillo & Rodríguez Fernández, 2011* (23) |  |  |  |  | + |  |  |  |  |  |  |  |  |  |  |  |  |  |  |  |  |
| *Chthonius (Ephippiochthonius) villacarrillo* Zaragoza & Pérez, 2013** (23) |  |  |  |  |  |  |  |  | + |  |  |  |  |  |  |  |  |  |  |  |  |
| *Chthonius* sp.** (#) |  |  |  |  |  |  |  |  |  |  |  |  |  |  |  |  |  |  | + | + |  |
| *Neobisium (Ommatoblothrus) espinoi* Carabajal Márquez, García Carrillo & Rodríguez Fernández, 2011** (23) |  |  |  |  | + |  |  | + |  |  |  |  |  |  |  |  |  |  |  |  |  |
| *Neobisium (Ommatoblothrus) perezruizi* Zaragoza & Pérez, 2013* (23) |  |  |  |  | + |  |  |  |  |  |  |  |  |  |  |  |  |  |  |  |  |
| *Neobisium (Ommatoblothrus) perezi* Carabajal Márquez, García Carrillo & Rodríguez Fernández, 2011* (23) |  |  |  | + | + |  |  |  |  | + | + |  |  |  |  |  |  |  |  |  |  |
| *Roncus boneti boneti* Beier, 1931* (2) |  |  |  |  |  |  |  |  |  |  |  |  | + |  |  | + |  |  |  |  |  |
| *Roncus boneti tarbenae* Mahnert, 1977* (2) |  |  |  |  |  |  |  |  |  |  |  |  |  |  | + |  |  |  |  |  |  |
| ARACHNIDA: ARANEAE | | | | | | | | | | | | | | | | | | | | | |
| *Centromerus* aff. *cavernarum* (L. Koch, 1872)** (26) |  |  |  |  |  |  |  |  | + |  |  |  |  |  |  |  |  |  |  |  |  |
| *Harpactocrates cazorlensis* Fernández, 1986** (26) |  |  |  |  |  | + |  |  |  |  |  |  |  |  |  |  |  |  |  |  |  |
| *Leptoneta comasi* Ribera, 1978* (17, 26) |  |  |  |  |  |  | + |  | + |  |  | + |  |  |  |  |  |  |  |  |  |
| *Lessertia barbara* (Simon, 1884)** (6, 26) | + |  |  | + |  | + |  |  |  |  |  |  |  |  |  |  |  |  |  |  |  |
| *Metellina merianae* (Scopoli, 1763)** (26) | + |  |  |  |  |  |  |  |  |  |  |  |  |  |  |  |  |  |  |  |  |
| *Centromerus paradoxus* (Simon, 1884)** (4) |  |  |  |  |  |  |  |  |  |  |  |  | + |  |  | + |  |  |  |  |  |
| *Cybaeodes magnus* Ribera & De Mas, 2015* (18) |  |  |  |  |  |  |  |  |  |  |  |  |  |  | + |  | + |  |  |  |  |
| *Dysdera espanoli* Ribera & Ferrández, 1986* (2) |  |  |  |  |  |  |  |  |  |  |  |  |  |  | + |  |  |  |  |  |  |
| *Eidmannella pallida* Emerton, 1875** (39) |  |  |  |  |  |  |  |  |  |  |  |  |  |  |  |  | + |  |  |  |  |
| *Lessertia dentichelis* (Simon, 1884)** (2) |  |  |  |  |  |  |  |  |  |  |  |  | + |  |  |  |  |  |  |  |  |
| *Leptyphantes fagei* Machado, 1939* (2) |  |  |  |  |  |  |  |  |  |  |  |  |  |  |  | + |  |  |  |  |  |
| *Leptyphantes zaragozai* Ribera, 1981* (2) |  |  |  |  |  |  |  |  |  |  |  |  |  |  | + |  |  |  |  |  |  |
| *Palliduphantes lorifer* (Simon, 1907)** (2, #) |  |  |  |  |  |  |  |  |  |  |  |  |  |  |  |  | + |  | + | + |  |
| *Palliduphantes gypsi* Ribera & De Mas, 2003** (26) |  |  |  |  |  |  |  |  |  | + |  |  |  |  |  |  |  |  |  |  |  |
| *Nesticus baeticus* López-Pancorbo & Ribera, 2011* (26) |  |  |  |  | + |  |  |  |  | + |  |  |  |  |  |  |  |  |  |  |  |
| CRUSTACEA: ISOPODA: ONISCIDAE | | | | | | | | | | | | | | | | | | | | | |
| *Anaphiloscia simoni* Racovitza, 1907** (1) |  |  |  |  |  |  |  |  | + |  |  |  |  |  |  |  |  |  |  |  |  |
| *Porcellio incanus* Budde-Lund, 1879** (1, 22) |  |  |  | + |  |  |  |  |  |  |  |  | + |  |  |  |  |  |  |  |  |
| *Eluma caelatum* (Miers, 1877)** (1) |  |  | + |  |  | + |  |  |  |  |  |  |  |  |  |  |  |  |  |  |  |
| *Porcellio nicklesi* Dollfus, 1892** (1) |  |  |  |  |  |  |  | + |  |  |  |  |  |  |  |  |  |  |  |  |  |
| *Trichoniscus* sp.** (1) |  |  |  | + |  |  |  |  |  |  | + |  |  |  |  |  |  |  |  |  |  |
| *Cristarmadillidium breuili* Vandel, 1954** (1, 2, 16) |  |  | + |  |  |  |  |  |  |  | + |  |  | + |  |  |  | + |  |  |  |
| *Cristarmadillidium muricatum* (Budde-Lund, 1885)** (2) |  |  |  |  |  |  |  |  |  |  |  |  |  |  |  |  | + |  |  |  |  |
| *Eleoniscus helenae* Racovitza, 1907* (2) |  |  |  |  |  |  |  |  |  |  |  |  |  |  | + |  |  |  |  |  |  |
| *Nesiotoniscus dianae* (Vandel, 1953)* (2, #) |  |  |  |  |  |  |  |  |  |  |  |  |  |  | + |  | + |  |  |  |  |
| *Parachaetophiloscia levantinus* Cruz & Dalens, 1989* (19) |  |  |  |  |  |  |  |  |  |  |  |  |  |  |  |  |  | + |  |  |  |
| *Porcellio bolivar* Dollfus, 1892** (16, #) |  |  |  |  |  |  |  |  |  |  |  |  |  | + |  |  |  | + |  |  |  |
| *Trichoniscu provisorius* Racovitza, 1908** (22) |  |  |  |  |  |  |  |  |  |  |  |  |  |  | + | + |  |  |  |  |  |
| *Trichoniscus perezi* García, 2008* (1) |  |  |  |  | + |  |  |  |  |  |  |  |  |  |  |  |  |  |  |  |  |
| MYRIAPODA: QUILOPODA: LITHOBIOMORPHA | | | | | | | | | | | | | | | | | | | | | |
| *Lithobius (Lithobius) castaneus* Newport, 1844** (28) |  |  |  |  |  | + | + |  |  |  |  |  |  |  | + |  |  |  |  |  |  |
| *Lithobius (Lithobius) erythrocephalus* C.L. Koch** (28, 40) |  |  |  | + |  | + |  |  |  |  |  |  |  |  |  |  |  |  |  |  |  |
| *Lithobius (Lithobius) lapidicola* Meinert, 1872** (28) |  |  |  |  |  |  |  |  | + |  |  |  |  |  |  |  |  |  |  |  |  |
| *Lithobius (Lithobius) motasi* Matic, 1967* (28) |  |  |  |  |  | + |  |  |  |  |  |  |  |  |  |  |  |  |  |  |  |
| *Lithobius (Monotarsobius) microps* Meinert, 1868** (28) |  |  |  |  |  | + | + |  |  |  |  |  |  |  |  |  |  |  |  |  |  |
| *Lithobius (Lithobius) pilicornis* Newport, 1844** (28) |  |  |  | + |  |  |  |  | + |  |  |  |  |  |  |  |  |  |  |  |  |
| MYRIAPODA: DIPLOPODA | | | | | | | | | | | | | | | | | | | | | |
| *Ceratosphys jabaliensis* Mauriès, 2013** (27) |  |  |  |  |  |  | + |  |  |  |  |  |  |  |  |  |  |  |  |  |  |
| *Ceratosphys solanasi* Mauriès & Vicente, 1975** (6, 27) | + | + |  | + | + |  |  |  |  |  |  |  |  |  |  |  |  |  |  |  |  |
| *Glomeris* sp.** (1) |  |  |  |  | + |  |  |  |  |  |  |  |  |  |  |  |  |  |  |  |  |
| *Lophoproctus jeanneli* (Brölemann, 1910)** (27) |  |  |  |  |  |  |  |  | + |  |  |  |  |  |  |  |  |  |  |  |  |
| *Macellolophus rubromarginatus* (Lucas, 1846)** (27) |  |  |  |  |  |  |  |  | + |  |  |  |  |  |  |  |  |  |  |  |  |
| *Polydesmus dismilus* Berlese, 1891** (2) |  |  |  |  |  |  |  |  |  |  |  |  | + |  |  |  | + |  |  |  |  |
| *Orimagtogona toniperezi* Mauriès, 2014** (3) |  |  |  | + |  |  |  |  |  |  |  |  |  |  |  |  |  |  |  |  |  |
| COLLEMBOLA | | | | | | | | | | | | | | | | | | | | | |
| *Acherontiella xenylliformis*** (20) |  |  |  |  |  |  |  |  |  |  |  |  |  |  |  |  |  |  |  | + |  |
| *Arrohopalites pygmaeus* (Wankel, 1860)** (20) |  |  |  |  |  |  |  |  |  |  |  |  |  |  |  |  |  |  | + |  |  |
| *Arrhopalites* sp. **(6) |  | + |  |  |  |  |  |  |  |  |  |  |  |  |  |  |  |  |  |  |  |
| *Deuteraphorura silvaria* (Gisin, 1952)** (29) |  |  | + |  | + |  | + |  |  | + | + |  |  |  |  |  |  |  |  |  |  |
| *Folsomia candida* (Willem, 1902)** (20, 29) |  |  |  |  |  | + |  |  |  |  |  |  |  |  |  |  |  |  | + |  |  |
| *Heteromurus nitidus* (Templeton, 1835)** (2, 6, 29) |  | + | + | + | + | + | + |  | + | + | + |  | + |  | + | + | + |  | + | + |  |
| *Lepidocyrtus flexicollis* Gisin, 1965** (29) |  |  |  | + | + | + |  |  | + | + | + |  |  |  |  |  |  |  |  |  |  |
| *Mesogastrura ojcoviensis* (Stach, 1918)** (20, 29) |  |  |  |  | + |  |  |  | + | + | + | + |  |  |  |  |  |  | + |  |  |
| *Mesachorutes quadriocellatus* Absolon, 1900** (29) |  |  |  | + | + |  |  |  |  |  |  |  |  |  |  |  |  |  |  |  |  |
| *Pseudosinella infrequens* Gisin & Da Gama, 1969** (20) |  |  |  |  |  |  |  |  |  |  |  |  |  |  |  |  |  |  | + |  |  |
| *Pseudosinella insularum* Dallai, 1969** (#) |  |  |  |  |  |  |  |  |  |  |  | + |  |  |  |  |  |  |  |  |  |
| *Pygmarrhopalites* sp.** (29) |  |  |  |  |  |  |  |  | + | + |  |  |  |  |  |  |  |  |  |  |  |
| *Pygmarrhopalites perezi* Arbea, 2013** (29) |  |  |  |  | + |  |  |  |  | + |  |  |  |  |  |  |  |  |  |  |  |
| *Gisinurus malatestai* Dallai, 1970** (16) |  |  |  |  |  |  |  |  |  |  |  |  |  | + |  |  |  |  |  |  |  |
| *Oncopodura* sp.** (16) |  |  |  |  |  |  |  |  |  |  |  |  |  | + |  |  |  |  |  |  |  |
| *Onychiurus ghidinii* Denis, 1938** (#) |  |  |  |  |  |  |  |  |  |  |  |  |  |  |  |  |  |  |  | + |  |
| *Pseudosinella templadoi* Simon & Selga, 1977** (#) |  |  |  |  |  |  |  |  |  |  |  |  |  |  |  |  |  |  |  | + |  |
| *Pseudosinella* sp.** (16) |  |  |  |  |  |  |  |  |  |  |  |  |  | + |  |  |  |  |  |  |  |
| *Sinella coeca* (Schott, 1896)** (21) |  |  |  |  |  |  |  |  |  |  |  |  |  |  |  |  |  |  | + |  |  |
| *Pseudosinella perezi* Arbea, 2013** (29) |  |  |  |  |  |  |  |  |  | + |  |  |  |  |  |  |  |  |  |  |  |
| *Troglopodetes absoloni* Bonet, 1931* (29) |  |  |  |  | + |  |  |  | + |  |  |  |  |  |  |  |  |  |  |  |  |
| DIPLURA | | | | | | | | | | | | | | | | | | | | | |
| *Cestocampa ibera* Sendra & Condé, 2012* (5) |  |  |  |  |  |  |  |  |  |  |  |  |  |  |  | + |  |  |  |  |  |
| *Metajapyx moroderi* (Silvestri, 1929)** (8, 9, 10) |  |  |  |  |  |  |  |  |  |  |  |  |  |  | + |  |  | + | + |  |  |
| *Plusiocampa lagari* Sendra & Condé, 1987* (6, 30) | + | + | + | + | + |  | + | + | + | + |  | + |  |  |  |  |  |  |  |  |  |
| *Plusiocampa lucenti* Sendra & Condé, 1986* (7) |  |  |  |  |  |  |  |  |  |  |  |  |  |  | + |  |  |  | + |  |  |
| INSECTA: ZYGENTOMA |  |  |  |  |  |  |  |  |  |  |  |  |  |  |  |  |  |  |  |  |  |
| *Coletinia diania* Molero, Bach & Gaju, 2013* (11) |  |  |  |  |  |  |  |  |  |  |  |  |  |  |  |  | + |  |  |  |  |
| *Coletinia intermedia* Molero, Bach & Gaju, 2013** (11) |  |  |  |  |  |  |  |  |  |  |  | + |  | + |  |  |  |  |  |  |  |
| *Coletinia* sp.** (33) |  |  |  |  |  |  |  |  | + |  |  |  |  |  |  |  |  |  |  |  |  |
| INSECTA: ORTOPTERA | | | | | | | | | | | | | | | | | | | | | |
| *Petaloptila (Zapetaloptila) mogon* Barranco, 2004** (12, 32) |  |  | + |  | + |  |  |  |  | + |  |  |  |  |  |  |  |  |  |  |  |
| *Petaloptila (Zapetaloptila) bolivari* (Cazurro, 1888)** (12) |  |  |  |  |  |  |  |  |  |  |  |  |  |  |  |  |  |  | + |  |  |
| *Petaloptila (Zapetaloptila) carabajali* Barranco, 2004** (12, 32) |  |  |  | + |  | + |  |  |  |  |  |  |  |  |  |  |  |  |  |  |  |
| *Petaloptila (Petaloptila) isabelae* Gorochov & Llorente, 2001** (6) |  | + |  |  |  |  |  |  |  |  |  |  |  |  |  |  |  |  |  |  |  |
| INSECTA: PSOCOPTERA |  |  |  |  |  |  |  |  |  |  |  |  |  |  |  |  |  |  |  |  |  |
| *Psyllipsocus ramburi* Selys-Longschamps, 1872** (37) |  | + |  |  | + |  | + |  |  |  |  |  |  |  |  |  |  |  | + |  | + |
| *Prionoglaris stygia* Enderlein, 1909** (6, 37) |  | + |  |  |  | + | + |  |  |  |  |  |  |  |  |  |  |  |  |  |  |
| INSECTA: COLEOPTERA: STAPHYLINIDAE | | | | | | | | | | | | | | | | | | | | | |
| *Atheta (Alaobia) tenebrarum* Assing, 2006** (12) |  |  |  |  |  | + |  |  |  |  |  |  |  |  |  |  |  |  |  |  |  |
| *Atheta (Alaobia) subcavicola* (Brisout de Barneville, 1863)** (#, 15) |  | + |  |  | + | + |  |  |  | + | + |  |  |  |  |  | + |  |  |  |  |
| *Atheta temeris* Assing & Vogel, 2003** (6) |  | + |  |  |  |  |  |  |  |  |  |  |  |  |  |  |  |  |  |  |  |
| *Domene lencinai* Vives, 2010** (6) |  | + |  |  |  |  |  |  |  |  |  |  |  |  |  |  |  |  |  |  |  |
| *Conosoma testaceum* (Fabricius, 1792)** (#) |  |  |  |  |  |  |  |  |  |  |  |  |  |  |  |  | + |  |  |  |  |
| *Domene perezi* Assing, 2012** (35) |  |  |  |  |  |  |  |  |  | + |  |  |  |  |  |  |  |  |  |  |  |
| *Sepedophilus cavicola* (Scriba, 1870)** (35) |  |  |  |  | + | + |  |  | + | + |  |  |  |  |  |  | + |  |  |  |  |
| INSECTA: COLEOPTERA: CARABIDAE | | | | | | | | | | | | | | | | | | | | | |
| *Laemostenus (Antisphodrus) cazorlensis cazorlensis* (Mateu, 1953)* (34) |  |  |  |  |  | + |  |  |  |  |  |  |  |  |  |  |  |  |  |  |  |
| *Laemostenus (Anthisphodrus) cazorlensis seguranus* (Vives & Vives, 1982)* (6, 34) | + | + |  |  |  |  | + |  |  | + |  |  |  |  |  |  |  |  |  |  |  |
| *Laemostenus (Laemostenus) complanatus* (Dejean, 1828)** (6) |  | + |  |  |  |  |  |  |  |  |  |  |  |  |  |  |  |  |  |  |  |
| *Laemostenus (Pristonychus) terrícola* (Herbst, 1783)** (2, 6, 34) | + | + |  |  |  |  |  |  |  |  |  |  |  |  | + |  | + | + |  |  |  |
| *Laemostenus (Pristonychus) baeticus* (Rambur, 1837) ** (34) |  |  |  |  | + |  | + |  | + |  |  |  |  |  |  |  |  |  |  |  |  |
| *Laemostenus (Anthisphodrus) levantinus* C. Bolivar, 1919* (#) |  |  |  |  |  |  |  |  |  |  |  |  |  |  |  |  |  |  | + |  |  |
| *Platyderus (Platyderus) breuili* Jeannel, 1921** (#) |  |  |  |  |  |  |  |  |  |  |  |  |  |  |  |  |  |  | + |  |  |
| *Porotachys bisulcatus* (Nicolaï, 1822)** (2) |  |  |  |  |  |  |  |  |  |  |  |  |  |  |  |  | + |  | + |  |  |
| *Trechus (Trechus) martinezi* Jeannel, 1927* (4) |  |  |  |  |  |  |  |  |  |  |  |  |  |  |  | + |  |  |  |  |  |
| *Trechus (Trechus) lencinai* Mateu & Ortuño, 2006* (6, 13) |  | + |  |  |  |  |  | + |  |  |  |  |  |  |  |  |  |  |  |  |  |
| *Trechus (Trechus) torressalai* Ortuño & Arillo, 2005* (39) |  |  |  |  |  |  |  |  |  |  |  |  |  |  |  |  | + |  |  |  |  |
| INSECTA: COLEOPTERA: LEIODIDAE |  |  |  |  |  |  |  |  |  |  |  |  |  |  |  |  |  |  |  |  |  |
| *Catops andalusicus* Heyden, 1870** (6, 36) |  | + |  |  |  | + | + |  |  |  |  |  |  |  |  |  |  |  |  |  |  |
| *Catops fuliginosus* Erichson, 1837** (36) |  |  |  |  |  | + |  |  |  |  |  |  |  |  |  |  |  |  |  |  |  |
| *Catops fuscus fuscoides* Reitter, 1909** (36) | + |  |  |  |  |  |  |  |  |  |  |  |  |  |  |  |  |  |  |  |  |
| *Catops nigricans* (Spence, 1815)** (36) |  |  |  |  |  | + |  |  |  |  |  |  |  |  |  |  |  |  |  |  |  |
| *Leptinus vaulogeri* Jeannel, 1922** (6, 36) |  | + |  |  |  | + |  |  |  |  |  |  |  |  |  |  |  |  |  |  |  |
| *Speonemadus angusticollis* (Kraatz, 1870)** (36) | + | + |  |  | + |  |  |  | + |  |  |  |  |  |  |  |  |  |  |  |  |
| *Speonemadus clathratus* (Perris, 1864)** (6) |  | + |  |  |  |  |  |  |  |  |  |  |  |  |  |  |  |  |  |  |  |
| *Speonemadus vandalitiae* (Heyden, 1870)** (6, 36) |  | + |  |  |  | + |  |  |  |  |  |  |  |  |  |  |  |  |  |  |  |
| *Anillochlamys bueni* Jeannel, 1909* (2) |  |  |  |  |  |  |  |  |  |  |  |  |  |  | + |  | + | + |  |  |  |
| *Anillochlamys tropica* (Abeille de Perrin, 1881)* (4) |  |  |  |  |  |  |  |  |  |  |  |  |  |  |  |  |  |  | + |  |  |
| *Spelaeochlamys ehlersi ehlersi* Dieck, 1870* (36) |  |  |  |  |  |  |  |  |  |  |  |  | + |  |  | + |  |  |  |  |  |
| INSECTA: COLEOPTERA: PTINIDAE | | | | | | | | | | | | | | | | | | | | | |
| *Ptinus (Ptinus) fur* Linnaeus, 1758** (6) |  | + |  |  |  |  |  |  |  |  |  |  |  |  |  |  |  |  |  |  |  |
| INSECTA: COLEOPTERA: SALPINGIDAE | | | | | | | | | | | | | | | | | | | | | |
| *Aglenus brunneus* Gyllenhal, 1813** (38, 39) |  |  |  |  |  |  |  |  | + |  |  |  |  |  |  |  | + |  | + |  |  |
| INSECTA: COLEOPTERA: CURCULIONIDAE | | | | | | | | | | | | | | | | | | | | | |
| *Otiorhynchus (Lixorrhynchus) torressalai* (Español, 1945)** (2) |  |  |  |  |  |  |  |  |  |  |  |  |  |  | + | + |  | + |  |  |  |
| INSECTA: HYMENOPTERA: FORMICIDAE | | | | | | | | | | | | | | | | | | | | | |
| *Aphaenogaster cardenai* Espadaler, 1981** (31) |  |  |  | + |  |  |  |  |  | + |  |  |  |  |  |  |  |  |  |  |  |

**References** Bibliographic references used to compile the biological inventories.

Ref. (1) Garcia, L. (2013) Isópodos terrestres (Crustacea: Oniscidea) recolectados en cavidades subterráneas de Jaén. Los Invertebrados de Hábitats Subterráneos de Jaén. (ed. by Pérez T. & A. Pérez,. eds.), pp. 78-85 G.E.V. Ed., Jaén.

Ref. (2) Zaragoza, J.A. & Sendra, A. (1988) Fauna cavernícola de la provincia de Alicante. Volumen III: Fauna, Flora, Ciencias y Medicina. (ed. by Diputación Provincial de Alicante) pp. 11-36. Ayudas a la Investigación 1984-1985. Instituto de Estudios Juan Gil-Albert. Alicante.

Ref. (3) Mauriès, J.P. (2014) Four new species of cavernicolous millipedes from Andalusia, Spain (Diplopoda: Polydesmida: Polydesmidae: Chamaesomatidae: Opisthocheiridae). Arthropoda Selecta, 23(1), 33-50.

Ref. (4) Sendra, A. & J.A. Zaragoza, 1982. Invertebrados cavernícolas del País Valenciano. Lapiaz, 10, 14-22.

Ref. (5) Sendra, A., Arnedo, M. A., Ribera, C., Teruel, S., Bidegaray-Batista, L. & Condé, B. (2012). Revision of *Cestocampa* Condé (Diplura, Campodeidae), with description of a new species from caves in the eastern Iberian Peninsula. Zootaxa, 3252, 43-56.

Ref. (6) Andújar, C, Pérez Hernández, T. & Lencina, J.L. (2015) Inventario de los invertebrados de cuevas del complejo subterráneo del Calar del Mundo (Riópar, Albacete). Gota a gota, 6, 87-93.

Ref. (7) Sendra, A., Lara, M.D., Ruiz Aviles, F. & Tinaut, A. (2004) Une nouvelle espèce du genre *Plusiocampa* Silvestri, 1912 (Diplura, Campodeidae) et données pour sa reconstruction paléobiogéographique dans les Bétiques. Subterranean Biology, 2, 113-122.

Ref. (8) Sendra, A., Ortuño, V.M., Moreno, A., Montagud, S. & Teruel, A. (2006) *Gollumjapyx smeagol* gen. n., sp. n., an enigmatic hypogean japygid (Diplura: Japygidae) from the eastern Iberian Peninsula. Zootaxa, 1372, 35–52.

Ref. (9) Silvestri, F. (1929) Contribución al conocimiento de los Japygidae (Thysan.) de España, Eos, 5, 81-95.

Ref. (10) Pagés, J. (1964) Remarques sur les Japygidae signales dans le domaine souterrain. International Journal of Speleology, 1(1,2), 191-201.

Ref (11) Molero, R, Bach, C., Sendra, A., Montagud, S., Barranco, P. & Gaju, M. (2013) Revision of the genus *Coletinia* (Zygentoma: Nicoletiidae) in the Iberian Peninsula, with descriptions of nine new species. Zootaxa, 3615(1), 1-60.

Ref. (12) Assing, V. (2006) Three new species of Staphylinidae from Spain, with a new synonymy (Insecta: Coleoptera). Linzer biologische Beiträge, 38(2), 1129-1137.

Ref (13) Ortuño, V. M. & Barranco, P. (2013) *Duvalius (Duvalius) lencinai* Mateu & Ortuño, 2006 (Coleoptera, Carabidae, Trechini) una especie hipogea del sur de la península ibérica. Morfología, reubicación taxonómica, sistemática y biología. Animal Biodiversity and Conservation, 36(2), 141-152.

Ref (14). Gonzalez, J.V. (1983) Distribució dels gastropods cavernicoles. Spélaion, 2, 53-54.

Ref (15). Lluch, R. (1986) Estafilinidae (Coleoptera) recolectados en cavidades subterráneas del País Valenciano. Lapiaz, 15: 32-33.

Ref (16). Sendra, A, Garay, P, Ortuño V. M., Gilgado J.D., Teruel, S., and Reboleira, A.S.P.S. (2014) Hypogenic versus epigenic subterranean ecosystem: lessons from eastern Iberian Peninsula. International Journal of Speleology, 43(3), 253-264.

Ref. (17) Ribera, C. (1978) *Leptoneta comasi* n. sp. (Araneae, Leptonetidae) una nueva especie cavernícola del Levante español. Miscel·lània Zoològica, 4 (2), 25-29.

Ref. (18). Ribera, C. & De Mas, E. (2015) Description of three new troglobiontic species of *Cybaeodes* (Araneae, Liocranidae) endemic to the Iberian Peninsula. Zootaxa, 3957, 313-323.

Ref. (19). Cruz, A. & Dalens, H. (1989) Especies nuevas o poco conocidas de isópodos terrstres de la Península Ibérica. I. Isópodos cavernícolas de la España oriental (Crustacea: Oinicidae). Bulletin de la Societé d'Histoire Naturelle de Toulouse, 125, 91-98.

Ref. (20). Gama, M.M. (2005) Collembola (Hexapoda) from caves of the Community of Valencia, Spain. Zoologica Baetica, 16, 47-57.

Ref. (21). Gama, M.M. (1984) Collemboles cavernicoles de l’Espagne I. Miscel·lània Zoològica, 8, 81-87.

Ref. (22) Cruz, A. (1991) Isópodos terrestres de la colección del Museu de Zoologia de Barcelona (Crustacea, Oniscidea). Miscel·lània Zoològica, 15, 81-102.

Ref. (23) Zaragoza, J.A. (2013) Los pseudoescorpiones (Arachnida) subterráneos de Jaén. Los Invertebrados de Hábitats Subterráneos de Jaén. (ed. by Pérez T. & A. Pérez), pp. 65-71. G.E.V. Ed., Jaén.

Ref. (24) Ríos, J. & Pérez, T. (2013) Los Moluscos (Gastropoda: Orthogastropoda) recogidos en las cavidades subterráneas de la provincia de Jaén. Los Invertebrados de Hábitats Subterráneos de Jaén. (ed. by Pérez T. & A. Pérez), pp. 24-30. G.E.V. Ed., Jaén.

Ref. (25) Subías, L.S. (2013) Oribáridos (Acari, Oribatida) subterráneos de Jaén. Los Invertebrados de Hábitats Subterráneos de Jaén. (ed. by Pérez T. & A. Pérez), pp. 56-57. G.E.V. Ed., Jaén.

Ref. (26) Ribera, C. (2013) Las arañas (Arachnida, Araneae) de las Cuevas de Jaén. Los Invertebrados de Hábitats Subterráneos de Jaén. (ed. by Pérez T. & A. Pérez), pp. 58-64. G.E.V. Ed., Jaén.

Ref. (27) Golovatch, S.I. & Maurés, J-P. (2014) Los diplópodos (Myriapoda, Diplopoda) subterráneos de Jaén. Los Invertebrados de Hábitats Subterráneos de Jaén. (ed. by Pérez T. & A. Pérez), pp. 88-93. G.E.V. Ed., Jaén.

Ref. (28) García, A. (2013) Los quilópodos (Myriapoda, Chilopoda) subterráneos de Jaén. Los Invertebrados de Hábitats Subterráneos de Jaén. (ed. by Pérez T. & A. Pérez), pp. 94-102. G.E.V. Ed., Jaén.

Ref. (29) Arbea, J.I. (2013) Los Colémbolos (Hexapoda, Collembola) subterráneos de Jaén. Los Invertebrados de Hábitats Subterráneos de Jaén. (ed. by Pérez T. & A. Pérez), pp. 104-114. G.E.V. Ed., Jaén.

Ref. (30) Sendra, A. (2013) Dipluros del medio subterráneo de Jaén. Los Invertebrados de Hábitats Subterráneos de Jaén. (ed. by Pérez T. & A. Pérez), pp. 115-116. G.E.V. Ed., Jaén.

Ref. (31) Tinaut, A. (2013) Los Formícidos (Insecta, Formicidae) de las cuevas de Jaén. Los Invertebrados de Hábitats Subterráneos de Jaén. (ed. by Pérez T. & A. Pérez,. eds.), pp. 124-12. G.E.V. Ed., Jaén.

Ref. (32) Barranco, P. (2013) Ortópteros (Insecta, Orthoptera) en cavidades de Jaén. Los Invertebrados de Hábitats Subterráneos de Jaén. (ed. by Pérez T. & A. Pérez), pp. 135-137. G.E.V. Ed., Jaén.

Ref. (33) Molero, R. & Gaju, M. (2013) Los Zigentomas (Insecta, Zygentoma) subterráneos de Jaén. Los Invertebrados de Hábitats Subterráneos de Jaén. (ed. by Pérez T. & A. Pérez), pp. 148-150. G.E.V. Ed., Jaén.

Ref. (34) Ortuño, V.M. & Pérez, T. (2013) La familia Carabidae en las cuevas de Jaén. Los Invertebrados de Hábitats Subterráneos de Jaén. (ed. by Pérez T. & A. Pérez), pp. 152-158. G.E.V. Ed., Jaén.

Ref. (35) Assing, V. (2013) Los estafilínidos registrados en cuevas de Jaén (Coleoptera, Staphylinidae). Los Invertebrados de Hábitats Subterráneos de Jaén. (ed. by Pérez T. & A. Pérez), pp. 159-163. G.E.V. Ed., Jaén.

Ref. (36) Fresneda, J. (2013) Los Leiódidos (Coleoptera, Leiodidae) subterréneos de Jaén. Los Invertebrados de Hábitats Subterráneos de Jaén. (ed. by Pérez T. & A. Pérez), pp. 164-175. G.E.V. Ed., Jaén.

Ref. (37) Pérez, T. (2013) Los psocópteros (Insecta, Psocoptera) subterráneos de Jaén. Los Invertebrados de Hábitats Subterráneos de Jaén. (ed. by Pérez T. & A. Pérez), p. 140. G.E.V. Ed., Jaén.

Ref. (38) Otero, J.C. & Pérez, T. (2013) Los Cryotophagidae, Latridiidae y Salpingidae (Coleoptera) subterráneos de Jaén. Los Invertebrados de Hábitats Subterráneos de Jaén. (ed. by Pérez T. & A. Pérez), pp. 176-178. G.E.V. Ed., Jaén.

Ref. (39) Ortuño, V.M. & Arillo, A. (2005) Description of a new hypogean species of the genus Trechus Clairville, 1806 from eastern Spain and comments on the Trechus martinezi‐lineage (Coleoptera: Adephaga: Carabidae). Journal of Natural History, 39(40), 3483-3500.

Ref. (40) García, A. (2016) Estado actual del conocimiento de los quilópodos cavernícolas de Jaén. Actas EspeleoMeeting Ciudad de Villacarrillo: 49-52.

**Figure S1.** Geostructral setting of the study area, the Prebaetic System, in the southeast of the Iberian Peninsula.

**
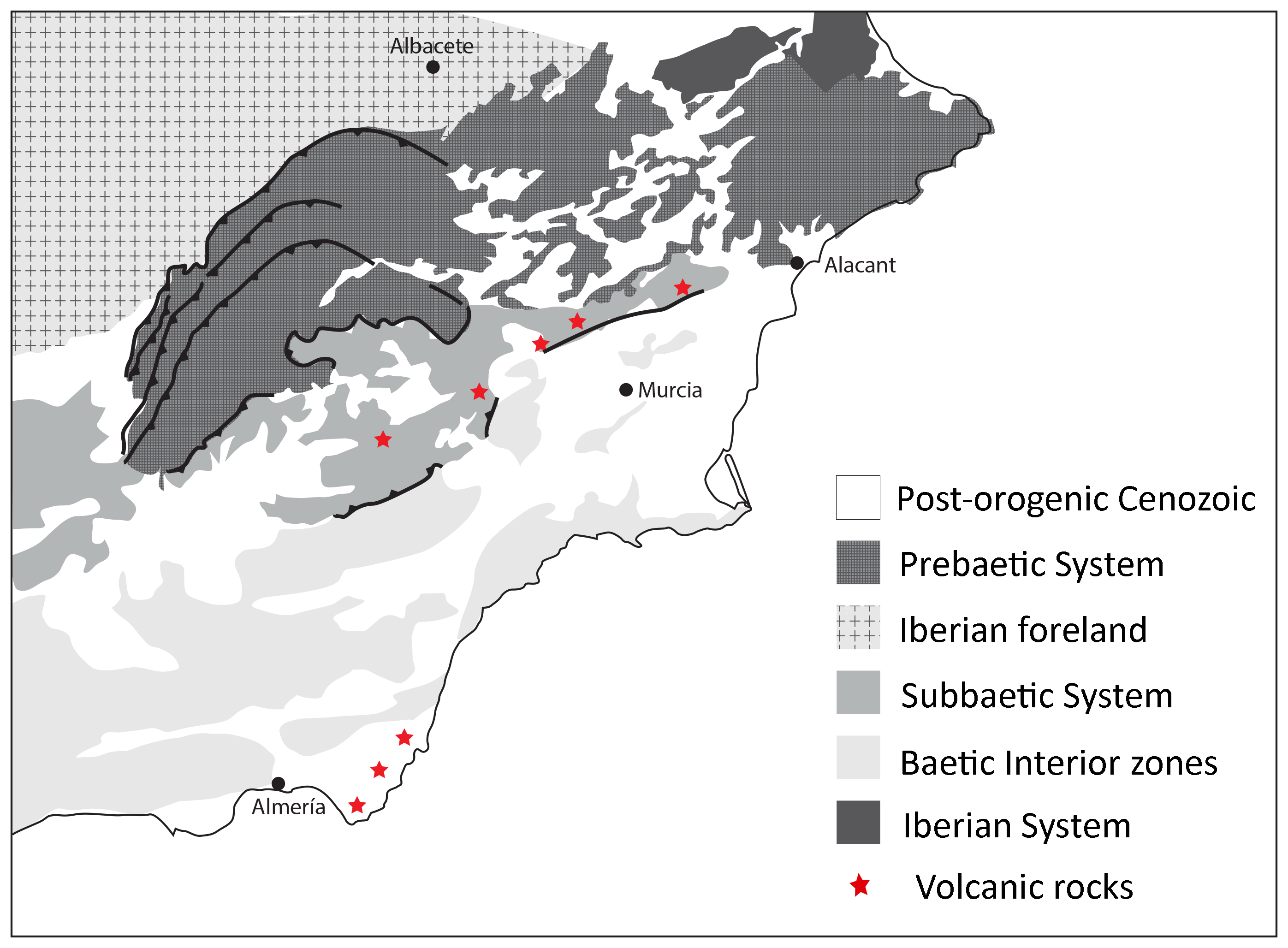
**

**Figure S2** Hotelling´s T^2^ values used to detect atypical observations in the PLSR analysis. Red line marks T^2^ critical value (4.55, p = 0.05).

**Figure S3** Results of the jackknife approach used to assess the robustness of the square weights of the three most relevant variables in the PLSR analysis. Red line represents the square weight above which a variable can be considered to have an effect higher than expected by chance (1/(number of response variables)). Dotted line represents the square weight of the variable using the 21 observations (see Table 2 in main text).

**Figure S4** Plots for the visual inspection of the assumptions of the LR model. Upper right, residuals against fitted values; upper left, normal qq-plot; lower right, scale-location plot; lower left, residuals against leverage plus Cook´s distance.

**Figure S5.** Geographical location and topography of the Puerto cave, and its three entrances (grey semicircles); the dashed yellow line delimits the contact zone between the dolomite limestone rock and the marls strata of the confining layer; the continuous line crossed by arrows shows the NW-SE anticline.


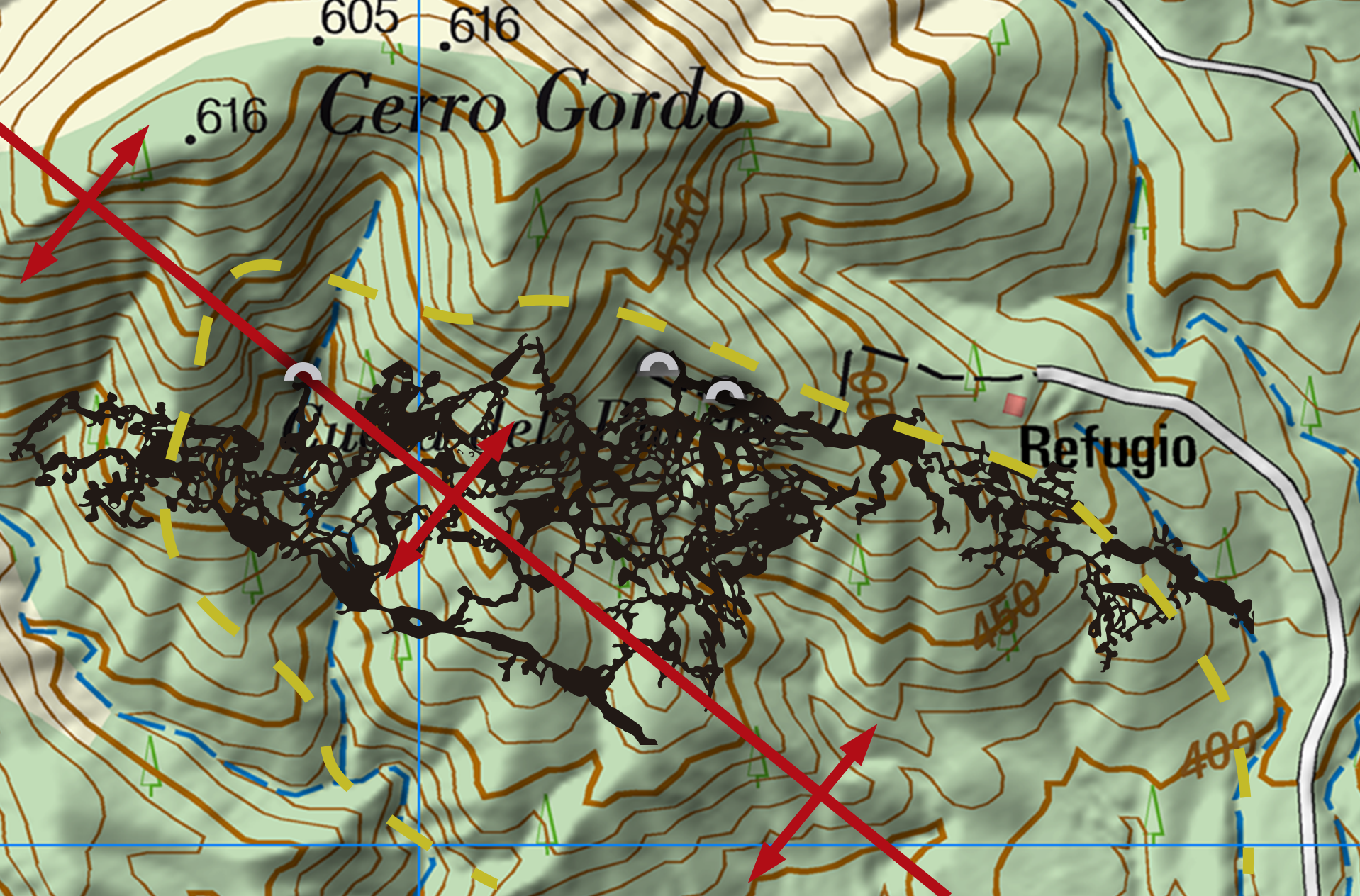


The Puerto cave shows clear geomorphological signs of hypogene speleogenesis (Ros *et al.* 2016). This 4 km-long cave is carved in dolomite-limestone rocks with thick layers that dip 30º NE. In the ceiling of this karstic carbonate rocks there are marl strata, which acted as a confining layer of the aquifer that produced the hypogene speleogenesis of the current cave. The ballooned morphology of the cave system is explained by the adaptation of the subterranean passages to the anticlinal position of the strata (Vera *et al.,* 1990). The speleogenetic period of the Puerto cave began when the confined aquifer was active, probably before and during the Inferior Quaternary. The disappearance of the marls, which acted as a confining layer, opened up the possibility for terrestrial fauna colonization much later (P. Garay, unpublished data). The Puerto cave has been explored by the GECA speleological group of Cieza (Murcia) from 1968 (GECA, 1970) after an entrance was opened during a mineral prospecting. Today, 4 kilometers of labyrinthic passages and chambers are known to reach down to a depth of 110 meters. In 2009, an artificial tunnel was created in another section of the cave, and some facilities were constructed to enable the Puerto cave to became a tourist attraction.

Vera Benito, C., Alcaraz Bastida, J.V. & Ruiz de Almirón, J.J. (1990). Topografía de cavidades con soporte informático. *Actas del V Congreso Español de Espeleología*: 24-28. Camargo, Santander.

GECA (1970). La Caverna del Puerto en Calasparra. *I Congreso Nacional de Espeleología*: 25-26. Barcelona.

Ros, A., Llamusí, J. L. & Sánchez, J. (2016). Morfologías hipogénicas en la Cueva del Puerto (Murcia, España). *Gota a Gota*, **10**: 8-15.
